# A longitudinal assessment of heat exposure and biomarkers of kidney function on heat shock protein 70 and antibodies among agricultural workers

**DOI:** 10.21203/rs.3.rs-3887323/v1

**Published:** 2024-01-24

**Authors:** Jaime Butler-Dawson, Richard J. Johnson, Lyndsay Krisher, Diana Jaramillo, Alex Cruz, Daniel Pilloni, Stephen Brindley, Bernardo Rodriguez-Iturbe, Laura Gabriela Sanchez-Lozada, Miranda Dally, Lee S. Newman

**Affiliations:** University of Colorado Anschutz Medical Campus; University of Colorado Anschutz Medical Campus; University of Colorado Anschutz Medical Campus; University of Colorado Anschutz Medical Campus; Grupo Pantaleón; Grupo Pantaleón; University of Colorado Anschutz Medical Campus; Instituto Nacional de Ciencias Médicas y Nutrición “Salvador Zubirán”; National Institute of Cardiology Ignacio Chávez; University of Colorado Anschutz Medical Campus; University of Colorado Anschutz Medical Campus

**Keywords:** Heat exposure, Kidney disease, Workers

## Abstract

**Background:**

Exposure to extreme heat impacts millions of people worldwide and outdoor workers are among the populations most affected by high temperatures. Heat stress induces several biological responses in humans, including the production of heat shock proteins (HSP) and antibodies against HSP (anti-HSP) which may play a central role in the body’s cellular response to a hot environment.

**Objective:**

This longitudinal study investigated the impact of high temperatures and humidity on the presence of HSP70 and anti-HSP70 and examined relationships with markers of kidney function in an at-risk workforce under conditions of extreme heat and exertion in Guatemala.

**Methods:**

We collected ambient temperature and relative humidity data as well as biomarkers and clinical data from 40 sugarcane workers at the start and the end of a 6-month harvest. We used generalized mixed-effects models to estimate temperature effects on HSP70 and anti-HSP70 levels. In addition, we examined trends between HSP70 and anti-HSP70 levels and markers of kidney function across the harvest.

**Results:**

At the end of the harvest, temperatures were higher, and workers had, on average, higher levels of HSP70 and anti-HSP70 compared to the beginning of the season. We observed significant increasing trends with temperature indices and HSP70 levels. Maximum temperature was associated with HSP70 increments after controlling for age, systolic and diastolic blood pressure (β: 0.21, 95% Confidence Interval: 0.09, 0.33). Kidney function decline across the harvest was associated with both higher levels of anti-HSP70 levels at the end of the harvest as well as greater increases in anti-HSP70 levels across the harvest.

**Conclusions:**

These results suggest that workplace heat exposure may increase the production of HSP70 and anti-HSP70 levels and that there may be a relationship between increasing anti-HSP70 antibodies and the development of renal injury. HSP70 holds promise as a biomarker of heat stress in exposed populations.

## Background

Exposure to extreme heat impacts millions of people worldwide.^1^ Outdoor workers are among the most vulnerable to health consequences of extreme heat.^2^ Heat stress induces a number of detrimental and defensive biological responses in humans. Further understanding of pathophysiologic responses has the potential for prevention through early detection and treatment. Among the known responses is increased production of heat shock proteins (HSP) and antibodies against HSP (anti-HSP) which contribute to release of proinflammatory cytokines, a potentially protective mechanism.^3^ The kidney is among the target organs of concern, based on the increasing evidence of a relationship between heat exposure and chronic kidney disease of unknown cause (CKDu).^4^ Few empirical human studies have examined the role of the HSP pathway or its involvement in the pathophysiology of heat-associated kidney injury under actual field conditions of extreme heat and exertion.

Outdoor workers, specifically agricultural workers, are exposed to various occupational hazards such as high temperatures and intense physical labor. High temperatures have increased considerably, and continue to increase, placing workers at risk for heat stress, which can result in heat illnesses, injuries and fatalities.^2^ There is increasing interest in the pathophysiologic role of endogenous proteins in response to meteorological conditions, including HSP. HSPs are synthesized in response to heat, inflammation, ischemia, and oxidative stress, as defending molecules that protect tissues from further injuries.^5^ In general, HSP levels are induced by naturally occurring stress and abnormal environmental conditions, to protect cells from damage due to these stresses. Thus, HSPs might be useful indicators of stress and stress responses. In addition, they may serve as a biomarker for evaluating disease states and the exposure response to environmental stressors among exposed workers and other at-risk community members.

Extracellular HSP as well as anti-HSP are not only sensitive biomarkers for heat stress but have been linked with renal inflammation and salt-sensitivity.^6^ Renal inflammation and systemic inflammation are hypothesized mechanisms underlying CKDu^7^ and there is emerging evidence that exposure to high temperatures and intense labor contribute to risk of kidney injury and CKDu among agricultural workers in Mesoamerica.^8–12^ HSPs play a role in kidney injury mechanisms because of their role in cellular inflammatory processes.^5^ Previously, we observed that workers who develop CKDu often have mild blood pressure elevations^13^ that could be a manifestation of the renal inflammatory response.^14^ The etiology and pathophysiology of CKDu remain poorly understood and HSPs may help us understand the response mechanisms to environmental stressors and their role in the development of CKDu.

Most HSPs are expressed under normal physiological conditions and, in general, act as chaperone molecules, mediating protein folding and transport.^15^ However, HSP levels rapidly rise in response to a wide variety of stresses such as exposure to high temperatures, environmental xenobiotics or chemical toxins and toxicants such as heavy metals, drugs, physiological stresses or autoimmunity. Furthermore, exposure to certain environmental stresses (including high temperatures and heavy metals) may also result not only in elevated HSP levels intracellularly but a release extracellular HSPs which stimulate an immune response by triggering the release of proinflammatory cytokines.^3^ Extracellular 70-kDa HSP (eHSP70) can mimic pathogenic antigens resulting in the development of autoimmunity with the creation of anti-HSPs.^5^ This occurs when HSPs present as self-antigens to the immune system, resulting in the production of autoantibodies to HSPs, which has been observed in patients with inflammatory diseases, autoimmune disorders and systemic arterial hypertension.^16, 17^ Additionally, autoimmunity to HSP can lead to chronic renal inflammation,^6, 18^ although there have been no known published studies of this potential pathway in research on the tubulointerstitial condition CKDu.

Our overarching hypothesis is that prolonged exposure to a combination of stresses including particulate matter and high temperatures may cause exposed workers to have high levels of eHSP70 and anti-HSP70. Because sugarcane and other agricultural workers along the Pacific coast of Central America have some of the highest reported rates of CKDu, we elected to investigate the potential relevance of eHSP70 and anti-HSP70, using blood samples and clinical data from a longitudinal cohort study of sugarcane workers laboring in intense heat during a 6-month harvest season in Guatemala. Our first aim was to cross-sectionally examine acute relationships between high temperature and high humid workplace environment and serum levels of eHSP70 and anti-HSP70 at the end of the work shift. Our second aim was to longitudinally examine relationships between serum eHSP70 and anti-HSP70 levels and biomarkers of renal function across the harvest which may serve as a biomarker for detection of early stages of CKDu among workers. We hypothesized that 1) higher temperatures across the work shift would lead to higher levels of eHSP70, 2) sugarcane workers at the end of the harvest, a period of higher environmental stressors, will have higher levels of circulating serum eHSP70 and anti-HSP70 than at the beginning of the harvest, a period of lower-environmental stress and, finally, 3) changes in markers of kidney function will be associated with levels of eHSP70 and anti-HSP70.

## Methods

### A. Study Design

This nested longitudinal study is part of a larger longitudinal study among adult male sugarcane cutters employed at a large agribusiness, Grupo Pantaleon (Pantaleon), in Southwest Guatemala during the 6-month harvest season from November 2017 to April 2018. This study was developed and conducted through a long-standing collaborative partnership with the company and its clinical staff under a memorandum of understanding with the University of Colorado. The study design and data collection procedures are outlined in detail in Butler-Dawson et al.^19^ In brief, 197 male sugarcane cutters were recruited from four different work groups at the start of the season in November 2017. Forty sugarcane cutters were randomly selected from the original study to include in the current study. Data collection occurred over two days in November, and at the end of the harvest over two days in April with the support of the clinical staff and locally hired research assistants. We collected meteorological data, venipuncture blood samples, urine specimens and clinical data at both time points. Blood and urine samples were collected at the end of the work shift. Meteorological indices were collected on the four study days from one local weather station within six kilometers of the fields. Mean and maximum temperature and humidity indices during the study day work shift hours were calculated. In addition, we measured serum creatinine and cystatin C to assess renal function, hemoglobin A1c (HbA1c) as an indicator of diabetes risk, serum uric acid because of observed associations with CKDu,^20^ and muscle injury using serum creatine kinase (CK). Work intensity was measured as the number of tons of cane each individual cut during the study work shift. Pantaleon shared data on each cane cutter’s daily amount of cane production.

Participants provided written informed consent at the time of recruitment. Clinical staff provided basic clinical results, abnormal kidney test results and hemoglobin A1c measures in the diabetic range to the participants in a private-setting with a culturally appropriate explanation and recommendation. IRB approval for this study was obtained from the Institutional Review Board of the University of Colorado and Comité de Ética Independiente ZUGUEME in Guatemala.

### B. Laboratory analysis

Serum eHSP70 and anti-HSP70 levels were measured in previously collected serum samples from the cutters. The serum samples were immediately frozen and stored in a freezer at the temperature of − 20°C before being transported frozen to our laboratory at the University of Colorado for long term storage at −70°C. We used commercial enzyme-linked immunosorbent assay (ELISA) kits to determine the levels of serum eHSP70 (DuoSet ELISA kits, DYC1663–05, R&D Systems, Minneapolis, MN, USA) and to determine the levels of anti-HSP70 (DuoSet ELISA kits), following the manufacturer’s instructions. Blood samples were analyzed at the time of venipuncture for all other serum tests. Serum creatinine was measured by automated standard techniques (Abbott, Architect CI4100) using kinetic alkaline picrate. Serum cystatin C values were determined by turbidimetry-based immunoassay. GFR) was estimated using the creatinine-based and cystatin C-based equations from Chronic Kidney Disease Epidemiology Collaboration (CKD-EPI) for all participants setting race to “non-Black”.^21^ Uric acid was measured via uricase with peroxidase and ascorbate oxidase (Abbott, Architect CI4100). CK was analyzed using CK-NAC serum start (DGKC) (Roche Cobas Integra 400 Plus). HbA1c was determined with ionic exchange high-pressure liquid chromatography (Biorad, D-10). Laboratory methods for the other biomarkers and clinical data are described in Butler-Dawson et al.^19^

### C. Statistical analysis

We summarized the clinical and biomarker variables by study visit with descriptive statistics. Results are presented as median and interquartile range (IQR) for continuous variables due to non-normal distributions based on the Shapiro–Wilk test. We evaluated differences between November and April clinical data and biomarkers using the non-parametric Wilcoxon signed rank sum test (paired data).

To assess acute relationships in serum eHSP70 and anti-HSP70 levels with weather indices and biomarkers across study work shift, we ran Linear Mixed Models (LMM) with a subject-specific random intercept regressing log-transformed serum eHSP70 and anti-HSP70 levels, separately, estimating β and 95% confidence intervals (95% CI). The repeated measures include the two time points. To identify independent predictors of serum eHSP70 and anti-HSP70 levels, multivariable analysis was performed. Variables with a p-value < 0.05 in univariate analysis were included in the multivariable model.

To examine cross-harvest relationships between eHSP70 and anti-HSP70 levels and markers of kidney function, we evaluated correlations by the Spearmen correlation coefficient between change in renal markers across the harvest and serum eHSP70 and anti-HSP70 levels. All analyses were completed in SAS version 9.4 (Cary, NC).

## Results

### Weather Indices.

The average temperature was 28.7°C and average humidity was 80.4% across the two study days in November with the maximum temperature reaching 31.8°C (Table 1). In April, the average temperature was 29.0°C and average humidity was 76.5% across the two study collection days, with the maximum temperature reaching 34.0°C.

### Clinical and Biomarker Data.

We included 40 participants in this study with data collected at the start and the end of the harvest, as presented in Table 1. The mean age was 33 years. We observed more hypertension in April as compared to November based on higher systolic blood pressure readings. Work intensity, measured by tons cut, was slightly higher in November compared to April but was not significantly different. We observed significantly higher CK levels in April compared to November.

### HSP70 and anti-HSP70.

**Figure 1:**
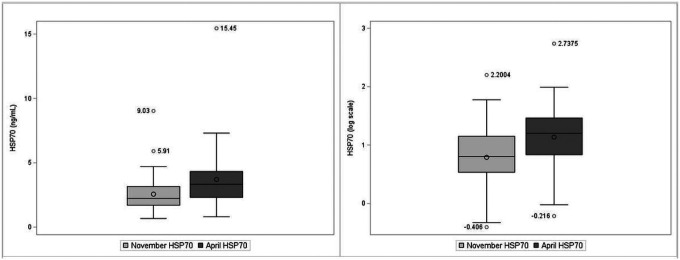
Box plots of heat shock protein 70 (HSP70) levels, ng/mL (left) and log-transformed HSP70 levels (right). Horizontal lines represent the median and boxes to the 25th–75th percentiles.

**Figure 2:**
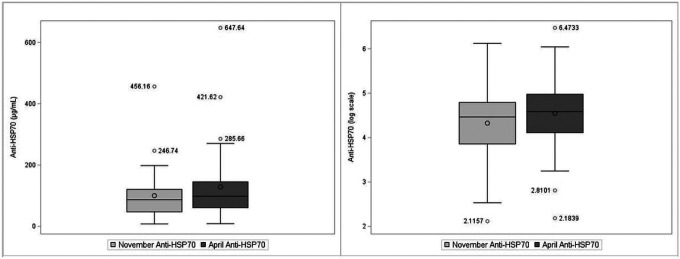
Box plots of antibodies against heat shock protein 70 (Anti-HSP70) levels, μg/mL (left), and log-transformed Anti-HSP70 serum levels (right). Horizontal lines represent the median and boxes to the 25th–75th percentiles..

At the start of the harvest in November (baseline), the median serum level of eHSP70 was 2.24 (interquartile range (IQR) 1.7, 3.16) ng/mL and, in April, was 3.33 (IQR 2.31, 4.32) ng/mL (Table 2). For serum anti-HSP70, the median level was 86.90 (IQR 47.34, 120.65) μg/mL in November and 98.31 (IQR 60.93–145.13) μg/mL in April. On average, serum eHSP70 levels in April were 1.1 ng/ml higher than baseline levels in November (p < 0.01). Similarly, the serum anti-HSP70 levels were 30.6 μg/mL higher in April as compared to November (p < 0.01). Distributions of HSP70 and anti-HSP70 and log transformed HSP70 and anti-HSP70 are shown in Figs. 1 and 2.

November serum eHSP70 levels were moderately positively correlated with April eHSP70 levels (r: 0.38; p = 0.02) and November serum anti-HSP70 levels were strongly positively correlated with April anti-HSP70 levels (r: 0.71; p < 0.01). We observed that cross-harvest change in eHSP70 was positively correlated with cross-harvest change in anti-HSP70 (r = 0.40; p = 0.01).

### A. Heat Exposure and HSP70 and anti-HSP70 levels

We conducted a repeated-measure analysis to investigate the acute associations between log-transformed serum eHSP70 and anti-HSP70 and meteorological conditions. In the univariate models, increasing mean and maximum temperatures were both associated with increasing serum eHSP70 (Table 3). Decreasing average humidity was associated with increasing serum eHSP70. In the multivariable model, maximum temperature remained associated with serum eHSP70 (β: 0.21, 95% CI: 0.09, 0.33) after controlling for age, systolic and diastolic blood pressure (Table 4). Decreasing uric acid levels were associated with increasing serum eHSP70 in the univariate model but this relationship was no longer significant in the multivariable model (β: −0.09, 95% CI −0.21, 0.03). We included maximum temperature as the one meteorological variable in the multivariable model, due to multicollinearity issues and consistency with the serum anti-HSP70 model (see below).

In the univariate models for anti-HSP70, increasing systolic blood pressure, maximum temperature, and eHSP70 were significantly associated with increases in anti-HSP70 levels. Decreasing average humidity was associated with increasing anti-HSP70 levels. In the multivariable model, eHSP70 (β: 0.067, 95% CI 0.01, 0.13) remained significant after controlling for age, systolic and diastolic blood pressure. HbA1c (β: −0.664, 95% CI: −1.34, 0.012) and maximum temperature (β: 0.001, 95% CI: −0.12, 0.12) were no longer significant. Of note, the hottest of the four days also had the lowest average humidity, with a maximum temperature of 34.0°C and a humidity of 71%, possibly explaining the observed opposite directions of the β estimates for temperature and humidity.

### B. Kidney function and eHSP70 and anti-HSP70 levels

Having reduced kidney function (eGFR-Cystatin C < 90 mL/min/1.73 m^2^) was more common in November than April (23% and 13%, respectively) with a similar trend with eGFR-Creatinine (Table 1). We did not see acute relationships in the repeated measures analysis between serum eHSP70 or anti-HSP70 levels with markers of muscle breakdown (CK) or markers of kidney function (eGFR-Creatinine and eGFR-Cystatin) at the end of the work shift (Table 3).

We examined correlations between eHSP70 and anti-HSP70 levels and markers of kidney function across the harvest. There was a moderate negative correlation between cross-harvest change in eGFR-Cystatin C and April anti-HSP70 levels (r = −0.49, p < 0.01) and cross-harvest change in anti-HSP70 levels (r= −0.38, p = 0.02). Negative cross-harvest change in eGFR-Cystatin C represents worsening in kidney function. Thus, worsening kidney function from November to April was correlated with higher levels of anti-HSP70 levels in April. Additionally, worsening kidney function from November to April was also correlated with increases in anti-HSP70 from November to April. We did not see significant correlations with cross-harvest change in eGFR-Creatinine. We performed post-hoc analyses based on these results to assess differences in anti-HSP70 levels between workers who had declines in eGFR-Cystatin C across the harvest (n = 15, change ranged from − 96.0 to −1.8) and workers who maintained or had increases in eGFR-Cystatin C across the harvest (n = 25, change ranged from 5.4 to 79.0). The 15 workers with declines across the harvest had significantly higher levels of April anti-HSP70 levels (median: 135.18 μg/mL; IQR: 105.48, 183.63) compared to the 25 workers who maintained or had increases in eGFR-Cystatin C (median: 72.3 μg/mL; IQR: 47.86, 109.30), Wilcoxon rank-sum test (p < 0.01). We also observed that those workers with declines in eGFR-Cystatin C across the harvest had greater increases in anti-HSP70 levels across the harvest (median change: 48.72; IQR: 20.87, 74.74) compared to workers who maintained or had increases in eGFR-Cystatin C (median change: 5.27; IQR: −25.61, 38.12), Wilcoxon rank-sum test (p = 0.04).

## Discussion

Under field conditions of high heat, humidity, and exertion, we have identified notable relationships that implicate eHSP70 and antibodies against HSP70 in the pathophysiologic response to extreme climatic and work conditions. Based on our repeated-measure analysis, high temperatures could be a key stress condition leading to higher levels of eHSP70 across the work shift. Furthermore, the data support the need for further investigation of the role of HSPs and their associated autoantibodies in the immunopathogenesis of CKDu, based on the observation associating anti-HSP70 levels with significant decline in eGFR-Cystatin C across the 6-month harvest. Measures of HSPs and autoantibodies may hold promise as biological markers of the human response to heat stress in the workplace, based on the observation that workers had higher levels of eHSP70 and anti-HSP70 at the end of the harvest during the hottest time of the season and the observation that maximum temperatures during the work shift were associated with higher eHSP70 level at the end of the work shift.

The elevation of eHSP70 levels in April could be a response to cumulative environmental stressors occurring from November to April, including, but not limited to, heat exposure. HSPs provide protection and accelerate repair of cells from heat exposure and other stressors. An increase in eHSP70 stimulates an immune response by triggering the release of proinflammatory cytokines.^3^ Previous studies have shown that many environmental factors within workplaces can increase levels of HSP70.^23, 24^ Our research supports the notion that high temperatures should be added to the list of environmental factors that lead to such elevations. Some evidence suggests that heat acclimatization increases baseline levels of eHSP70.^25^ At the cellular level, heat acclimatization includes adaptations that make cells more tolerant to heat shock. This process is known as acquired thermal tolerance and is associated with HSPs.^26^ It has been suggested that it is possible that the accumulation of intracellular HSP70 during heat acclimatization is dependent on repeated elevations in core temperature but there is limited data to support these claims.^27^ HSP synthesis begins within hours of heat exposure and could be dependent on the imposed cumulative heat,^26^ which is supported by this current study’s findings. HSPs are likely important modifying factors in an individual’s response to a variety of physiologically relevant conditions, such as heat and other stressors.^26^ While we did see higher levels of CK in April compared to November, we did not see univariate relationships between creatine kinase and eHSP70; further research is needed to understand the role of muscle injury and cell lysis in relation to HSP. Nevertheless, exercise and heat stress induce HSP70 overexpression in other organs, such as liver and brain which might contribute to increases in eHSP70.^25, 28^

In this study, we also measured anti-HSP70 levels and observed relationships with change in eGFR-Cystatin C across the 6-month harvest. While we did see acute associations with temperature in the univariate models, in the multivariable model the only factor that was signi cant was eHSP70. HSPs can stimulate the immune system, and antibodies against HSP70 are likely produced in response to elevation of HSP70. Anti-HSP70 can be found under normal physiological conditions, after exposure to environmental stresses, and in many diseases.^17, 28^ Wu et al. (1996) have shown that steel industry workers who had long-term exposure to high temperatures, carbon monoxide, and other chemicals in coke ovens have antibodies to HSP27, HSP60, HSP70, and HSP90 in plasma.^23^ Another study by Wu et al. showed that workers with exposure to heat, dust, benzene, and noise have increased frequency of anti-HSP70.^29^ Interestingly, experimental data suggest that the presence of autoantibodies against HSP augments the production of proinflammatory cytokines by human macrophages.^30^

The possible significance of HSPs and/or anti-HSPs in heat-induced illnesses and diseases, such as CKDu, is unknown. While we didn’t observe acute relationships between eHSP70 and markers of kidney function, we observed higher levels of anti-HSP70 among workers who had declines in kidney function across the harvest compared to workers who did not have declines in kidney function. Although our study does not directly test the relationship between HSP and HSP antibodies and proinflammatory cytokine production and cannot establish a causal link to the tubulointerstitial inflammation seen in CKDu, the lines of evidence suggest that the connection to the high inflammatory state observed in renal disease merits further research. Our research contributes to the limited number of studies on HSP molecules in renal diseases. Chebotareva et al. (2018) showed that anti-HSP70 serum levels were significantly higher in patients with nephrotic syndrome (21.1, IQR 17.47–29.72 pg/ml) than in controls (18.9, IQR 13.5–23.9 pg/ml).^28^ Musial et al. (2010) found that anti-HSP70 was elevated in children with CKD, although serum HSP70 levels did not differ from those of controls.^31^ In another study, authors observed higher HSP70 levels in patients with chronic glomerulonephritis^32^ and that HSP60 mRNA and protein levels were elevated in proximal tubular cells after heat stress.^33^ HSP70 has been shown to activate a chronic low-grade inflammatory state while simultaneously having a protective effect against oxidative stress and inflammation.^28^ Our data suggest the need for further research relating HSP and its antibodies to the inflammatory pathway that may lead to heat-associated disorders including the tubulointerstitial disorder CKDu.

Our research has a number of limitations. We considered this to be a small-scale preliminary study which calls for replication in a research study with a larger cohort and more time points. As a largely correlational study, we cannot establish causal relationships between temperature, kidney function, and HSP and anti-HSP. In addition, elevated eHSP70 and anti-HSP70 levels may be associated with other stress conditions that we did not measure. We did not directly examine the relationship between HSP and anti-HSP and markers of inflammation, leaving examination of this element of the hypothesized pathogenic pathway for future research. Other highly conserved HSP moieties may be important but were not measured in our study. Environmental measures of heat and humidity exposures were obtained from a local weather station six kilometers from the fields. More proximal, microclimatic conditions may give a more precise estimate of actual exposures. In addition, we did not directly measure body temperature in the cohort on the study days. A direct measure of proximal heat and humidity, core body temperature, and biomarkers of heat stress would strengthen the links suggested by our data.

## Conclusions

We conclude that exposure to high temperatures in the workplace may contribute to increased expression of HSP70. We offer lines of evidence connecting high working temperatures, elevations of HSP70 and anti-HSP70, and deterioration of renal function across the harvest season. Future studies are warranted to determine the role of thermally induced endogenous protein production in the pathogenesis of CKDu.

## Figures and Tables

**Table 1:** Summarized statistics of worker characteristics, biomarkers, and weather indices.

	November (baseline), n = 40	April (end of season), n = 40	P-value
**Demographics and Clinical**			
Age, years, mean ± SD	33 ± 11	-	-
HbA1c, %, mean ± SD	5.6 ± 0.4	Not measured	-
SBP, mmHg, mean ± SD	107 ± 9	116 ± 12	< 0.01*
DBP, mmHg, mean ± SD	72 ± 8	68 ± 10	0.01*
- HTN, n (%)	0 (0%)	3 (8%)	-
Tons of cane cut, median (IQR)	5.6 (5.0, 6.2)	5.4 (4.0, 6.2)	0.25
**Biomarkers, median (IQR)**			
Serum Uric Acid, mg/dL	5.84 (4.85, 6.57)	5.40 (4.80, 6.29)	0.20
Serum Creatine Kinase, units/L	260.0 (217.0, 379.5)	321.0 (248.0, 463.0)	0.03*
Serum Creatinine, μmol/L	77.81 (69.85, 93.73)	76.93 (68.97, 91.96)	0.64
Serum Cystatin C, mg/L	0.75 (0.53, 0.94)	0.59 (0.48, 0.79)	0.03*
eGFR-Creatinine, ml/min/1.73m^2^	110.62 (88.82, 121.58)	108.41 (97.35,118.08)	0.77
- < 90, n (%)	10 (25%)	6 (15%)	< 0.01*
eGFR-Cystatin C, mi/min/1.73m^2^	118.13 (92.35, 146.21)	136.43 (113.2, 150.41)	0.03*
- < 90, n (%)	9 (23%)	5 (13%)	0.25
**Weather Indices, 2-day average [day 1, day 2]**			
Mean Humidity, %	80.4 [84.2, 76.6]	76.5 [81.9, 71.1]	-
Maximum Humidity, %	100.0 [100.0, 100.0]	100.0 [100.0, 100.0]	-
Mean Temperature, °C	28.7 [28.2, 29.1]	29.0 [28.0, 30.0]	-
**Demographics and Clinical**			
Maximum Temperature, °C	31.6 [31.4, 31.8]	32.9 [31.7, 34.0]	-

SD, standard deviation. IQR, interquartile range. SBP, systolic blood pressure. DBP, diastolic blood pressure. HTN, Hypertension was defined as systolic blood pressure ≥ 140 mmHg or diastolic blood pressure ≥ 90 mmHg. eGFR, estimated glomerular filtration rate. P-values were obtained by paired Wilcoxon signed-rank test.

* Indicates significance at p < 0.05.

**Table 2:** Summarized statistics for extracellular heat shock protein 70 (eHSP70) and antibodies to HSP70 (anti-HSP70) levels.

	November (baseline), n = 40	April (end of season), n = 40	P-value	Change from November to April*
eHSP70, ng/mL, median (IQR)	2.24 (1.71, 3.16)	3.33 (2.31, 4.32)	0.01	Mean ± SD: 1.14 ± 2.70
				Median (IQR): 0.98 (−0.11, 2.29)
Anti-HSP70, Mg/mL, median (IQR)	86.90 (47.34, 120.65)	98.31 (60.93, 145.13)	0.01	Mean ± SD: 30.56 ± 67.96
				Median (IQR): 16.04 (−12.33, 53.83)

IQR, interquartile range. SD, standard deviation. P-values between November and April values were obtained by paired Wilcoxon signed-rank test.

* Positive change represents higher values in April.

**Table 3:** Results from univariate linear mixed-effect models examining associations between serum extracellular Heat Shock Protein 70 (eHSP70), and antibodies to HSP70, (anti-HSP70) and weather indices and markers of kidney function.

	eHSP70 Models		Anti-HSP70 Models	
	Estimate^A^ (95% CI)	p-value	Estimate^A^ (95% CI)	p-value
Clinical				
Age	0.01 (−0.01, 0.02)	0.47	0.001 (−0.02, 0.03)	0.92
HbA1c	0.24 (−0.16, 0.64)	0.24	−0.628 (−1.27, 0.01)	0.05
Systolic	0.01 (−0.004, 0.02)	0.20	0.015 (0.01, 0.03)	< 0.01*
Diastolic	−0.01 (−0.02, 0.01)	0.47	0.0001 (−0.01, 0.01)	0.99
Biomarkers				
Uric Acid	−0.12 (−0.23, −0.001)	0.048*	−0.138 (−0.28, 0.01)	0.07
Creatine Kinase	0.0003 (−0.0003, 0.001)	0.36	0.00038 (−0.0002, 0.001)	0.21
eGFR-Creatinine	−0.001 (−0.01, 0.01)	0.83	0.001 (−0.01, 0.01)	0.88
eGFR-Cystatin C	0.002 (−0.002, 0.01)	0.28	−0.002 (−0.01, 0.0)	0.20
eHSF70	-		0.085 (0.03, 0.14)	< 0.01*
Weather Indices				
Mean Humidity	−0.05 (−0.08, −0.02)	< 0.01*	−0.032 (−0.06, 0)	0.04*
Mean Temperature, °C	0.31 (0.12, 0.5)	< 0.01*	0.16 (−0.02, 0.34)	0.08
Maximum Temperature, °C	0.18 (0.08, 0.27)	< 0.01*	0.091 (0.01, 0.17)	0.03*

A Coefficient of log-transformed HSP70 and anti-HSP70 concentrations.

* Indicates significance at p < 0.05.

**Table 4:** Results from multivariable linear mixed-effect models examining associations between serum extracellular Heat Shock Protein 70, (eHSP70), and antibodies to HSP70, (anti-HSP70), and weather indices and markers of kidney function.

	eHSP70 Model		Anti-HSP70 Model	
	Estimate ^A^ (95% CI)	p-value	Estimate ^A^ (95% CI)	p-value
Age, years	0.01 (−0.01, 0.02)	0.48	0.004 (−0.02, 0.03)	0.75
Systolic BP mmHg	−0.002 (−0.02, 0.01)	0.76	0.01 (−0.01, 0.02)	0.16
Diastolic BP mmHg	0.01 (−0.01, 0.02)	0.44	−0.004 (−0.02, 0.01)	0.69
Maximum Temperature, °C	0.21 (0.09, 0.33)	< 0.01*	0.009 (−0.13, 0.11)	0.99
Uric Acid, mg/dL	−0.09 (−0.21, 0.03)	0.12	-	
eHSP70, ng/mL	-		0.065 (0.01, 0.13)	0.03*

A Coefficient of log-transformed HSP70 and anti-HSP70 level.

* Indicates significance at p < 0.05.

## Data Availability

The dataset analyzed during the current study is available from the corresponding author on reasonable request.
